# Gait and Conditioned Fear Impairments in a Mouse Model of Comorbid TBI and PTSD

**DOI:** 10.1155/2018/6037015

**Published:** 2018-09-20

**Authors:** Peyton Teutsch, Carolyn E. Jones, Mara E. Kaiser, Natasha Avalon Gardner, Miranda M. Lim

**Affiliations:** ^1^VA Portland Health Care System, Portland, OR, USA; ^2^Department of Behavioral Neuroscience, Oregon Health & Science University, Portland, OR, USA; ^3^Portland State University, Portland, OR, USA; ^4^Department of Neurology, Department of Medicine, Division of Pulmonary and Critical Care Medicine, Oregon Institute of Occupational Health Sciences, Oregon Health & Science University, Portland, OR, USA

## Abstract

**Study Objectives:**

Traumatic brain injury (TBI) and posttraumatic stress disorder (PTSD) commonly cooccur. Approaches to research and treatment of these disorders have been segregated, despite overlapping symptomology. We and others have hypothesized that comorbid TBI + PTSD generates worse symptoms than either condition alone. We present a mouse model of comorbid TBI + PTSD to further explore this condition.

**Methods:**

A mouse model of TBI + PTSD was generated using the single prolonged stress (SPS) protocol in combination with the controlled cortical impact (CCI) protocol. This resulted in four experimental groups: control, TBI, PTSD, and TBI + PTSD. Behavioral phenotyping included gait analysis, contextual fear conditioning, acoustic startle response, and prepulse inhibition.

**Results:**

Mice in the TBI + PTSD group showed a significantly impaired gait compared to their counterparts with TBI alone as well as control mice. Mice in the TBI + PTSD group showed significantly impaired contextual fear recall compared to controls. Prepulse inhibition testing revealed intact acoustic startle and auditory sensory gating.

**Conclusions:**

These results indicate that SPS paired with CCI in mice produces unique behavioral impairments in gait and fear recall that are not present in either condition alone. Further studies are underway to examine additional behavioral, physiological, and pathological phenotypes in this combined model of TBI + PTSD.

## 1. Introduction

Traumatic brain injury (TBI) is a change in neural functioning caused by an external force to the head, resulting in mild to severe injury [1]. TBI results in persistent symptoms including changes in cognition (e.g., difficulty concentrating or remembering), problems with balance, and increased anxiety. TBI is highly prevalent, resulting in approximately 2.5 million yearly diagnoses [2], with an alarming occurrence in the Veteran community of nearly 20% of Veterans reporting a diagnosed TBI [3].

Posttraumatic stress disorder (PTSD) can develop after a traumatizing event and frequently results in behavioral changes that overlap in symptomatology with TBI, including cognitive dysfunction, heightened anxiety, and exaggerated startle response [4]. Compared to the average population, rates and symptom severity of PTSD are reported to be significantly higher in Veterans [5]. Both TBI and PTSD are associated with lower quality of life and increased risk for psychological and medical disorders compared to healthy individuals [6]. Furthermore, Veterans with TBI in particular are at a higher risk for developing PTSD [6].

The comorbidity of TBI and PTSD in the same individual may present a unique symptomology; the extent of which has yet to be fully explored and may depend on the severity of either condition. Conventional approaches to research and treatment of these disorders in the human population have been segregated, despite overlapping symptomology. Thus, the aim of this study was to characterize behavioral outcomes in a mouse model of TBI + PTSD to add to the development and characterization of this emerging field of research [7–10]. We hypothesized that the combination of these two conditions would harbor its own distinct phenotype compared to controls or either condition alone. We used the well-established models of single prolonged stress (SPS) [11] to create a mouse model of PTSD and controlled cortical impact (CCI) [12] as a model of TBI, in order to examine the combined TBI + PTSD behavioral phenotype. Behavior testing reported here includes digital gait analysis, contextual fear conditioning, acoustic startle, and auditory prepulse inhibition.

## 2. Materials and Methods

### 2.1. Animals

Male, C57Bl/6J wild-type mice (The Jackson Laboratory), 8-9 weeks in age and weighing 23–30 g, were used for this study. Mice were housed in groups of four and were allowed to acclimate to the colony after shipment for one week prior to beginning the study. Housing was in a 12 : 12 h light : dark cycle (lights on at 0700 h) with ad libitum access to food (LabDiet® PicoLab® Laboratory Rodent Diet, 5L0D) and water. Temperature (21.6°C) and humidity (24%) were monitored and maintained at a constant level throughout the experiment. All protocols and housing were approved by the Institutional Animal Care and Use Committee at the Portland VA and adhered to guidelines set forth by the National Institutes of Health Guide for Care and Use of Laboratory Animals.

### 2.2. Experimental Design

**Figure 1:**
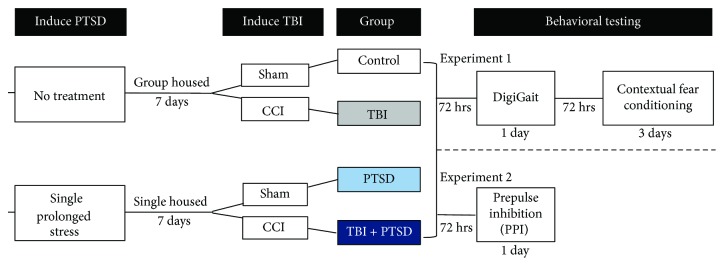
Experimental design and timeline. Subjects were randomized to SPS or no treatment. Mice were then further randomized to receive either a CCI or sham surgery to result in our four studied groups: control, TBI, PTSD, and TBI + PTSD. Behavioral testing in experiment 1 began 72 hours after surgeries with one day of gait testing, then 72 hours later began three days of contextual fear conditioning. Experiment 2 consisted of 1 day of PPI testing 72 hours after surgeries.

Mice were randomized into one of four groups (control, TBI, PTSD, and TBI + PTSD; see Figure 1 for design). TBI was generated using the controlled cortical impact (CCI) model, and PTSD was generated using the single prolonged stress (SPS) protocol. Owing to the methodological complexity of SPS, the SPS protocol was performed first, followed by CCI. Two experiments were conducted: in experiment 1, mice underwent gait testing and contextual fear conditioning and in experiment 2, a separate cohort of mice underwent acoustic startle and prepulse inhibition testing (Figure 1).

### 2.3. Single Prolonged Stress (SPS)

Since its initial development in rats [11], SPS has successfully been applied to other rodent models, including mice [13–16]. Briefly, SPS consists of a sequential series of stressors: tube restraint, forced swim, ether anesthesia, and finally social isolation for one week [11]. Half of the mice in all experiments were randomized to receive the SPS protocol. For tube restraint stress, mice were placed into ventilated 50 mL conical vials, which were then placed in a clean cage with bedding, for two hours. After tube restraint, groups of four mice were immediately placed together into a single plastic tub (8.5 × 9.0 × 12.0 in) filled with room temperature water (26°C) deep enough to prevent tails from touching the bottom (three-quarters full). Group forced swim lasted a duration of 20 minutes, during which time the mice and water temperature were closely monitored. Following the group forced swim, mice were towel dried and placed into individual bell jars containing a cotton ball soaked with 1.0 mL of diethyl ether. Mice were carefully monitored and immediately removed from the jar once they lost consciousness. Subsequently, mice were placed in a standard home cage to be individually housed for seven days of social isolation. Each SPS cohort began at 0800 h and ended with individual housing at 1300 h. Mice that were not randomized to SPS remained group housed for the duration of seven days that their counterparts underwent social isolation. Social isolation is a key component of SPS, and it has been shown to be a necessary incubation period for this trauma model [14, 17].

### 2.4. Controlled Cortical Impact (CCI)

Experimental mice were randomized to either a CCI or a sham surgery condition. In combination with SPS above, this resulted in four groups in total (control, TBI, PTSD, and TBI + PTSD). For CCI, mice were weighed, anesthetized using isoflurane (3% to induce, 1% to maintain), and secured to a stereotaxic frame (Stoelting Company). Mice had their heads shaved and sterilized using alcohol and iodine, before topical lidocaine was applied to the shaved head. An incision was made on the right side of the prepared skin to expose the skull landmarks bregma and lambda, and 30% hydrogen peroxide was used on a cotton applicator to remove the membranes covering the skull. Using a hand drill (Fine Science Tools), a 3 mm craniotomy was made to the right of the midline, between bregma and lambda. An impactor arm (Kopf Instruments) was then used to deliver an impact at a depth of 3.0 mm and velocity of 0.5 m/s, with a dwell time of 0.1 s to the cortical surface. The scalp incision was sutured, and triple antibiotic ointment was applied to the incision site. Mice were given a 1.0 mL subcutaneous injection of saline and returned to a standard home cage on a circulating warming pad with access to food, water, nestlets, DietGel® 76A (ClearH_2_O®), and 1.0 mL children's cherry flavored acetaminophen (Q-Pap) in 15 mL of water. Mice in the sham surgery condition underwent anesthesia and scalp incision, followed by application of hydrogen peroxide to the skull and immediate closure of the incision with sutures. All mice were housed individually following surgery for the remainder of the study to ensure recovery from surgery.

### 2.5. Gait Testing

**Figure 2:**
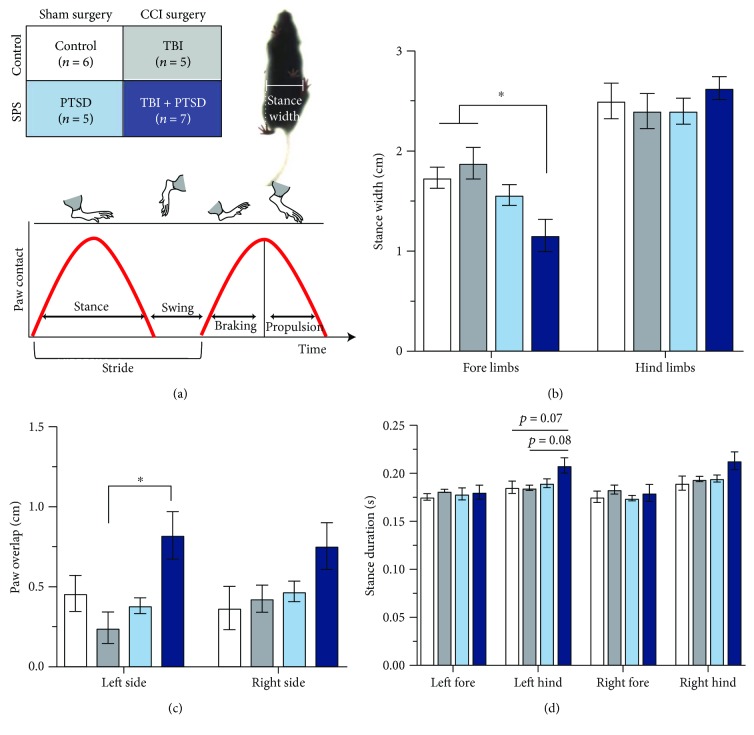
Gait metrics after TBI + PTSD. (a) Experimental design and diagram of the mouse gait representing a step cycle and stance width measures. (b) Stance width in fore and hind limbs. Forelimb stance width was decreased in TBI + PTSD mice compared to TBI alone (*p* = 0.008) and controls (*p* = 0.029). (c) Paw overlap in left and right sides. Paw placement in the left side of TBI + PTSD mice was significantly increased compared to TBI alone mice (*p* = 0.018). (d) Stance duration in each paw. Stance duration in the left hind limbs was significant in the omnibus ANOVA (*p* = 0.046), and post hoc testing was trending in TBI + PTSD relative to control and TBI alone mice. Error bars ± SEM, ^∗^*p* < 0.05.

Mice were tested for differences in gait using the DigiGait™ imaging system (Mouse Specifics). The DigiGait system consisted of a transparent treadmill belt surrounded by a Plexiglas compartment (17.4 cm × 5.1 cm × 13.3 cm) that was illuminated and filmed from below by a high-speed imaging video camera shot at 150 frames/s. Mice were given one minute to acclimate to the treadmill testing chamber, then were run for ~5 s at a belt speed of 25 cm/s. Mice were excluded for failure to walk on the treadmill (*n* = 2), failure to walk at least six consecutive strides (*n* = 2), and for technical difficulties of dim lighting affecting usable video acquisition (*n* = 4 per group). Mice were then removed from the treadmill chamber and returned to their home cage. All testing occurred between 0830 and 1000 h. Gait was analyzed using DigiGait imaging software (version 14) according to manufacturer's instructions by a scorer blinded to the group to interpret metrics involving stance and other indices of gait. For the purpose of this study, we focused on measures of stance (e.g., stance duration—time the paw spent in contact with the treadmill belt) and stability (stance width and paw placement positioning) since TBI has been found to affect stance and stature in humans [18]. DigiGait metrics were provided for both fore and hind paws on both sides. Individual values for a priori determined stance and paw placement metrics were analyzed for each paw. A schematic of gait metrics is shown in Figure 2(a).

### 2.6. Contextual Fear Conditioning

Contextual fear conditioning and recall was conducted over three days in the first four hours of the light cycle (0830 h–1100 h). Behavior was recorded with digital cameras (GoPro Hero Session) that were watched by a trained observer blind to the experimental group and scored manually for freezing upon completion of the experiment. Freezing was defined as the cessation of movement with the exception of respiration. In test sessions, total time freezing over a two-minute period was scored with a stopwatch program (Stopwatch+, Georgia State University) and expressed as a percentage of total time spent freezing. This time period was chosen to examine contextual fear memory recall only, while avoiding engaging fear extinction mechanisms. Scoring of freezing behavior began 20 s after placement into the chamber.

#### 2.6.1. Fear Conditioning Day 1: Contextual Fear Acquisition (Context A)

Two identical testing chambers (40.8 cm × 14 cm × 18.4 cm, Omnitech Electronics) were used for fear conditioning. One animal was excluded from fear conditioning due to equipment failure with the shock delivery system. Fear conditioning chambers consisted of four clear acrylic walls and a clear top for overhead video recording. Each chamber had a metal floor that consisted of 45 stainless steel rods spaced 5 mm apart (context A). The unconditional stimulus (US) was a 1.0 mA scrambled footshock (duration = 1 s). Fear conditioning and testing occurred in acoustic isolation boxes configured with a light and fan that provided ambient noise at 65 dB. Mice were placed in the chamber and after three minutes received 5 US deliveries with a fixed intertrial interval (ITI) of 60 s. Mice were removed immediately after the termination of the last shock; as such, there was no freezing measure collected after delivery of the final US.

#### 2.6.2. Fear Conditioning Day 2: Context Generalization (Context B)

In order to test contextual discrimination and generalization of fear memories, mice were returned to the fear conditioning boxes the following day with changes in contextual cues from the prior acquisition day. Walls were lined with black and white checkered paper on three of the four walls; a smooth black floor insert covered the shock bars, and a lemon-scented cleaning wipe was used on the pull-out waste tray (context B). Additionally, mice were placed in a different chamber location than the first day of fear conditioning. Mice remained in this new context for two minutes. During this time, no US was delivered.

#### 2.6.3. Fear Conditioning Day 3: Fear Recall Test (Context A)

**Figure 3:**
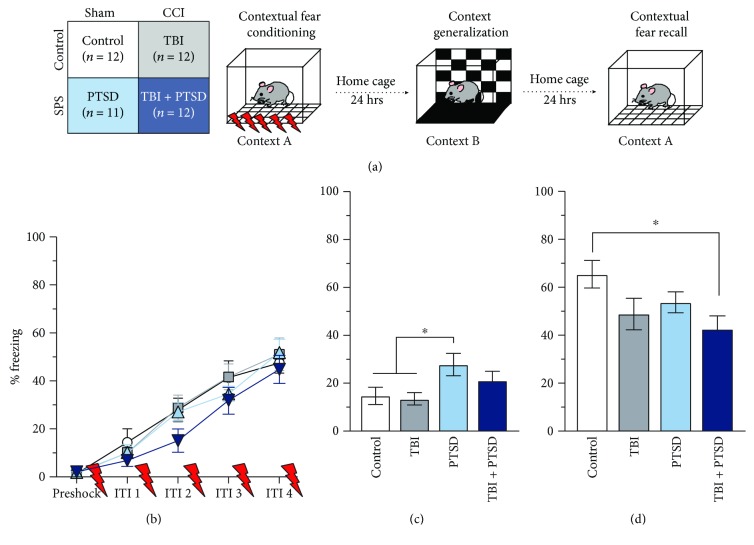
Impaired fear recall in TBI + PTSD mice following contextual fear conditioning. (a) Experimental design and fear conditioning procedure. (b) Contextual fear acquisition. There were no group differences in fear learning. (c) Context generalization. PTSD mice froze significantly more in context B compared to both control (*p* = 0.012) and TBI only mice (*p* = 0.010). (d) Contextual fear recall. TBI + PTSD mice showed deficits in contextual fear recall relative to controls (*p* = 0.033). Error bars ± SEM, ^∗^*p* < 0.05. ITI = intertrial interval.

The testing chambers were reverted to context A; the same context was used for fear acquisition—clear walls, floor comprised of parallel metal rods, and no additional scent. A fear recall test was performed by placing mice in the feared context and scoring freezing behavior for two minutes. No US was delivered during this time. A schematic of the contextual fear conditioning protocol is shown in Figure 3(a).

### 2.7. Acoustic Startle and Prepulse Inhibition (PPI)

**Figure 4:**
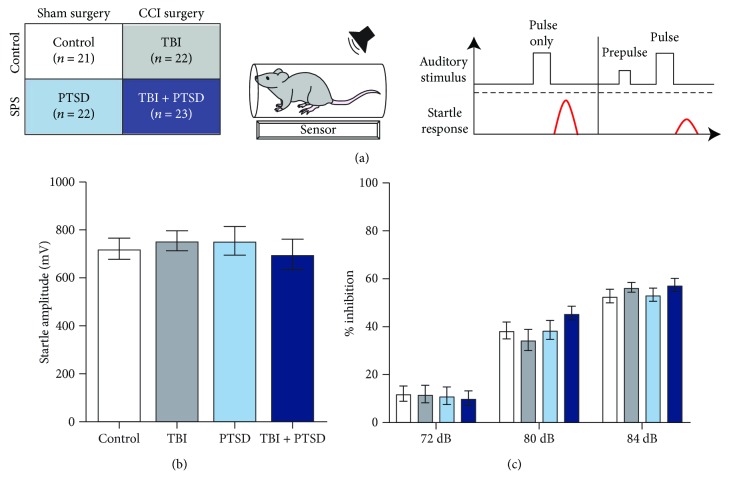
Comorbid TBI + PTSD does not affect acoustic startle response or prepulse inhibition. (a) Schematic of experimental design, acoustic testing chamber, and startle protocol. (b) Acoustic startle response. There were no changes in acoustic startle response across groups when the 120 dB pulse was played without a prepulse. (c) Prepulse inhibition. There was a significant interaction between prepulse level and treatment group on inhibition of the startle response to the pulse; however, there were no group differences in overall PPI. Background noise was constantly generated at 65 dB. Error bars ± SEM. dB = decibel.

In experiment 2, the same design of TBI and PTSD was examined for acoustic startle reflex and PPI. Following SPS and/or a CCI depth of 2.5 mm, mice were placed in isolation boxes inside a clear acrylic tube affixed over a piezoelectric accelerometer (San Diego Instruments) that captured animal movement in response to precisely calibrated white noises of varying intensity emitted from a loudspeaker. For each trial, a testing box was left empty, acting as an ambient sensor to capture any background noise that may offer interference to the collected readings. At no point did the ambient sensor register movement higher than that of a box containing an animal. Testing began 5 minutes after mice were placed in the chambers. The test consisted of 66 trials divided into 11 blocks. Each block consisted of null trials with background 65 dB noise only, startle only trials consisting of the pulse (120 dB) alone, and prepulse trials of 72, 80, or 84 dB preceding a 120 dB pulse (fixed interstimulus interval (ISI) = 100 ms). Trial order within each block was pseudorandomized so that no trial type occurred consecutively, and trials were separated by a variable 15 s ITI (maximum ITI = 20 s, minimum ITI = 10 s). All testing occurred between 0900 and 1100 h. A schematic of acoustic startle and PPI apparatus is shown in Figure 4(a).

### 2.8. Data Analysis

Statistical analyses were performed using one-way analysis of variance (ANOVA) with the treatment group (control, TBI, PTSD, and TBI + PTSD) as the between-subject factor. Significant main effects were explored with Tukey HSD post hoc tests. If measures were taken from animals at repeated time points (PPI testing and contextual fear acquisition), a repeated measures ANOVA was used. When data was not normally distributed, the Kruskal-Wallis nonparametric test was conducted and post hoc comparisons were performed using Dunn's test to follow up significant main effects. Analyses were conducted in IBM SPSS (version 24.0) with figures produced in GraphPad Prism (version 7.0). Results were considered significant at an alpha value of 0.05. All data are represented as means ± standard error of the mean (SEM).

## 3. Results

### 3.1. Gait Testing

In the forelimbs, stance width was altered (one-way ANOVA, *F*(3,21) = 5.38, *p* = 0.007), such that the TBI + PTSD mice showed significantly decreased stance width compared to both the TBI group (*p* = 0.008) and the control group (*p* = 0.029), with all other *p* values > 0.207 (Figure 2(b)). Paw placement positioning was altered in the left side (Kruskal-Wallis, *p* = 0.028), with differences emerging between the TBI + PTSD group and the TBI group (*p* = 0.018) (Figure 2(c)). There was also a significant main effect of the group on stance duration in the left hind paw (one-way ANOVA, *F*(3,19) = 3.209, *p* = 0.046), with a trend towards increased stance duration in TBI + PTSD mice relative to controls (*p* = 0.072) and TBI mice (*p* = 0.084), with all other *p* values > 0.214 (Figure 2(d)). Between groups, mice did not differ in weight at the time of gait testing (one-way ANOVA; *F*(3,26) = 0.303, *p* = 0.828) (Table 1).

### 3.2. Contextual Fear Conditioning

All groups acquired contextual fear as evidenced by increased freezing across the acquisition session (repeated measures ANOVA, within subjects: *F*(3,111) = 66.843, *p* < 0.0001), and there were no differences between groups (repeated measures ANOVA, between subjects *F*(3,37) = 0.946, *p* = 0.428) as well as no interaction (repeated measures ANOVA, trial × group interaction, *F*(9,111) = 0.425, *p* = 0.919) (Figure 3(b)). Two animals were excluded from analysis during acquisition because of video camera malfunction. Mice did not display a fear response when first placed in a fear conditioning context (freezing prior to shock onset was less than 2% for all groups, Figure 3(b)).

Generalization of fearful memories was determined by altering the contextual cues associated with the fear conditioning chamber and measuring freezing for a two-minute period. There was a significant main effect of the group on freezing in the new context (Kruskal-Wallis, *p* = 0.029), where PTSD mice froze significantly more than both TBI alone (*p* = 0.015) and controls (*p* = 0.017) (Figure 3(c)).

Fear recall was tested by returning the mice to the fear conditioning context for two minutes and measuring freezing. There was a significant main effect of the group on fear recall as measured by freezing to the fear conditioned context (one-way ANOVA, *F*(3,43) = 2.965, *p* = 0.042). TBI + PTSD mice froze significantly less than controls (*p* = 0.033) during the contextual fear recall test (Figure 3(d)).

### 3.3. Acoustic Startle and Prepulse Inhibition

Baseline startle to a 120 dB pulse is shown in Figure 4(b). There were no group differences in average startle response to the 120 dB pulse alone (one-way ANOVA, *F*(3,84) = 0.273, *p* = 0.845).

Prepulse inhibition was determined by comparing percent startle inhibition between groups and prepulse decibel levels. Percent startle inhibition was calculated by dividing the average startle value across each prepulse amplitude by the average startle across trials that were not preceded by a prepulse (pulse alone trials). The following formula was used to calculate percent inhibition at each decibel level: [(1 − (average prepulse startle/average pulse alone startle)) × 100]. Repeated measures ANOVA with the dB level of prepulse (70 dB, 80 dB, and 84 dB) as the within-subject factor and treatment group as the between-subject factor revealed, as expected, a significant within-subject effect of the PPI level with increasing amplitudes of prepulses leading to increased inhibition of the startle response to the subsequent pulse (repeated measures ANOVA, within subjects: *F*(2, 168) = 491.153, *p* < 0.0001). There was a prepulse level × group interaction (*F*(6,168) = 2.411, *p* = 0.029) but no between-group effect (*F*(3,84) = 0.366, *p* = 0.778) (Figure 4(c)).

## 4. Discussion

We found that mice in the TBI + PTSD group showed a significantly impaired gait compared to mice with TBI alone and controls. Mice in the TBI + PTSD group also showed significantly impaired fear recall compared to controls. Prepulse inhibition testing revealed no overall effect of TBI, PTSD, or combined TBI + PTSD on baseline acoustic startle and sensory gating ability. Taken together, these results indicate that SPS paired with CCI in mice produces unique behavioral impairments in gait and fear recall that are not present in either condition alone.

### 4.1. TBI + PTSD Impairs Gait

Gait is frequently examined in animal models of TBI, likely owing to damage to the motor circuits that control gait caused by CCI and other common TBI protocols. As of now, there are no standardized parameters for TBI induced by CCI and research has shown that variations in speed and/or depth of impact can influence both cognitive and motor outcomes differentially [19]. In our model of TBI, the injury was administered to the right hemisphere of the brain and our results showed predominantly contralateral effects on gait.

In this model of TBI + PTSD, we found that stance width in the forelimbs was dramatically decreased and paw placement position, a measure that indicates the amount of ipsilateral overlap between fore and hind paws, was preferentially increased on the left side in the TBI + PTSD mice. Paw overlap may reflect the ability of mice to balance and may contribute to the instability seen in the decreased forelimb stance width in the TBI + PTSD mice. In humans, narrower stance widths tend to be unstable [20], suggesting that gait alterations in TBI + PTSD mice contribute to unstable walking patterns. Human subjects with TBI have been found to have an increased stance time [18] and often complain of balance problems and difficulty walking, especially when multitasking [21, 22]. The clinical observation that TBI patients have trouble multitasking may provide insight to our results. In our data, we observed distinctive gait impairments only in mice in the TBI + PTSD group. This is consistent with the noted phenomenon in which deficits in TBI in human patients are brought out or worsened with stress or increased cognitive load [21, 23]; the addition of PTSD as a form of chronic or ongoing stress induces gait deficits in TBI mice that are otherwise masked with TBI alone.

TBI alone was not sufficient to induce gait changes, in contrast to what others have found in other CCI mouse models of TBI [24, 25]. In these models, gait changes seem to only emerge after repeated exposure to the treadmill, which we did not incorporate into our protocol [24, 25]. One possibility is that a single test on the DigiGait apparatus may not be complex enough to elicit changes in the TBI only group, whereby postinjury gait impairments may only emerge in subjects with the cognitive burden of PTSD.

Other studies using TBI alone found effects on stance duration that ranged from being present in all paws [24] to only being seen in the contralateral forelimb [25]. In keeping with our findings in the TBI alone group, other studies where mice were also only given one chance to use the treadmill, Luo and colleagues [26] did not observe significant changes in a repetitive, closed-head induced TBI [26]. It is possible that repeated gait testing is more sensitive to detecting gait deficits associated with TBI in rodents. Initially, we chose not to perform gait training and repeated testing due to the injury model of CCI and the timing of its pairing with SPS, as the prolonged length of time required to run SPS would also prolong the length of time elapsed since TBI.

To be able to definitively understand what factors are influencing gait, future studies will look at gait testing in larger cohorts of animals and with altered parameters, such as treadmill training. This will help determine if injury from the TBI is responsible for the effect or if other conditions are driving our results.

### 4.2. TBI + PTSD Impairs Contextual Fear Recall

In addition to changes in gait, we sought to investigate the pathological fear and startle response characteristic of PTSD within this combined TBI + PTSD mouse model. Overgeneralization of fear responses is common in patients with PTSD, presumably owing to their inability to differentiate between dangerous and neutral contexts [27, 28]. Patients with PTSD may also not be able to use environmental cues to modulate fear expression in the appropriate context [29]. Using contextual fear conditioning, where a previously neutral context is paired with an aversive event (e.g., footshock) such that later exposure to the context elicits a fear response (e.g., freezing), we confirmed that mice that underwent SPS showed increased fear generalization when placed in a new context after associative fear learning, consistent with overgeneralized fear responses present in patients with PTSD [27–29] as well as mouse models of PTSD [30]. Although we hypothesized that mice that had undergone SPS would show increased freezing during fear recall (when returned to context A), we did not observe an increase. One possibility is that the short context B test served as a brief extinction session for the SPS mice potentially reducing the freezing displayed in the following 24 hours.

Additionally, we found that mice with combined TBI + PTSD showed deficits in contextual fear recall 48 hours after contextual fear conditioning as evidenced by decreased freezing when first returned to the feared context. It will be important in future research to determine if this deficit in memory recall is specific to contextual fear learning as well as memory of nonaversive events. We did not find differences during the contextual fear acquisition session, which suggests that fear learning and the expression of freezing are intact in all of our groups. The fear recall deficit is specific to retrieval of the contextual fear memory 48 hours after conditioning and possibly represents impaired memory systems within the combined TBI + PTSD model.

Importantly, our observed contextual fear memory impairment was found only in the combined TBI + PTSD mice and not in mice that received a TBI alone. This suggests that the injury itself is not the cause of the memory impairment. Future research will expand on these results to examine how a contextual fear memory, acquired postinjury, changes over an extended period of time using this model to further explore memory impairments in TBI or TBI + PTSD animals. Sierra-Mercado et al. (2015) also used the CCI method to induce TBI in mice, examined auditory cued fear conditioning, and found no deficits in conditioning, recall, or extinction in CCI mice. Although we used a purely contextual model of fear learning, our TBI results are similar even though our test was conducted only one-week postinjury versus two weeks in the study by Sierra-Mercado et al. Additionally, our results add to data obtained by Ojo et al. [8], who failed to see any differences in spatial learning or memory recall in a TBI group alone but did observe auditory cued fear memory impairments in their model of TBI + PTSD [8]. Our data show that combined TBI + PTSD reduces freezing 48 hours after contextual fear conditioning and suggest that the combined condition impairs contextual fear memory recall. This is consistent with the mixed literature and continues to add to the variable results of fear learning after trauma in animal models.

### 4.3. Acoustic Startle Response and Prepulse Inhibition Are Intact

We anticipated that mice in the PTSD groups would have an increased baseline acoustic startle response, as has been reported in patients with PTSD [31]. However, we did not observe an increased acoustic startle response in mice in the PTSD groups when pulse trials (120 dB) were presented alone. Our findings are consistent with another study of veterans with PTSD [32], which found no change in acoustic startle response relative to controls. It has been theorized that these inconsistent results in subjects with PTSD may be attributed to differences in symptom severity [33].

Given the phenomenology of human subjects with PTSD, we sought to measure sensory gating in our mouse model of TBI and PTSD using prepulse inhibition testing. Prepulse inhibition tests the phenomenon of sensory gating whereby the occurrence of a low intensity “prepulse” milliseconds before the onset of a high intensity, startle inducing, “pulse,” leads to a dampening of the physiological startle response to the pulse. If successful prepulse inhibition has occurred, the recorded startle response would be lower than in the absence of the prepulse. The timing of these trials is on the order of milliseconds and is not consciously detectable to the subject. Importantly, the sensory gating phenomenon and ability to test it with prepulse inhibition testing equipment are conserved across species and altered in some psychiatric conditions.

In human subjects with PTSD, results are mixed regarding the ability to appropriately filter out irrelevant auditory stimuli in tests of prepulse inhibition, with some report finding and others failing to see differences in individuals with PTSD compared to control populations [34]. Here, we add to the growing body of literature investigating sensory gating and the baseline acoustic startle response in PTSD. In our hands, the SPS model of PTSD does not alter startle or prepulse inhibition responses either on its own or in conjunction with a CCI model of TBI, in mice. Taken together, these results suggest that the basic brain circuits underlying both acoustic startle and sensory gating ability remain intact in this population. However, the treatment groups in our study influenced sensory gating changes at different prepulse levels, despite no overall group effects on prepulse inhibition. In our studies, we used a standard 100 ms delay between the prepulse and pulse. TBI work in mice also using the CCI model has found similar interaction effects that become more pronounced at longer delays between the prepulse and pulse (100 ms and 200 ms) [35]. Interestingly, when they shortened the delay to 50 ms, there were no differences in sensory gating ability [35]. This suggests that factors such as the extent of injury and difficulty of a given task are key to consider when interpreting results in animal models of TBI and PTSD [35].

## 5. Conclusion

Here, we describe a unique model of TBI + PTSD in laboratory mice that recapitulates some of the behavioral impairments found in the patient population with these disease states. By combining single prolonged stress with controlled cortical impact, we have developed a controlled laboratory model of TBI + PTSD. Mice with comorbid TBI + PTSD present with unique impairments in their gait profile and problems with memory recall after contextual fear conditioning without affecting acoustic startle response or their ability to gate sensory stimuli.

Further studies are underway to examine additional behavioral, physiological, and pathological phenotypes in this combined model of TBI + PTSD. As we begin to develop an understanding of how TBI + PTSD symptoms manifest in humans, the use of this animal model to further explore the underlying physiological changes associated with combined TBI + PTSD will be invaluable in both the diagnosis and treatment of this patient population.

## Figures and Tables

**Table 1:** Mouse weights at DigiGait testing. There were no weight differences between groups at the time that DigiGait testing was conducted (*p* = 0.823).

Group	Weight (g)	SD	*N*
Control	28.19	1.55	8
TBI	27.91	2.51	7
PTSD	28.47	2.5	8
TBI + PTSD	28.89	1.09	7

## Data Availability

The data used to support the findings of this study are available from the corresponding author upon request.
